# Successful Treatment of Cutaneous Squamous Cell Carcinoma Complicated by Myelodysplastic Syndrome Using Combined ALA‐PDT and Narrow‐Margin Excision: A Case Report

**DOI:** 10.1002/ccr3.72256

**Published:** 2026-03-10

**Authors:** Yunwei Yang, Zhenlin Li, Long Wen, Yongxian Lai, Guolong Zhang

**Affiliations:** ^1^ School of Medicine Anhui University of Science and Technology Huainan China; ^2^ Department of Phototherapy, Shanghai Skin Disease Hospital, School of Medicine Tongji University Shanghai China; ^3^ Skin Cancer Center, Shanghai Skin Disease Hospital, School of Medicine Tongji University Shanghai China; ^4^ Institute of Photomedicine, School of Medicine Tongji University Shanghai China

**Keywords:** ALA‐PDT, cutaneous squamous cell carcinoma, local narrow‐margin excision, myelodysplastic syndromes, non‐melanoma skin cancer

## Abstract

Cutaneous squamous cell carcinoma (cSCC) is the second most common type of non‐melanoma skin cancer. When the cSCC patient is complicated by myelodysplastic syndromes (MDS), treatment becomes particularly challenging due to immunosuppression, bleeding risk, and poor tolerance to conventional therapies. Here, we report a case of a 77‐year‐old woman with cSCC and MDS who was successfully treated by a combination of narrow‐margin surgical excision and 5‐aminolevulinic acid photodynamic therapy (ALA‐PDT). No recurrence was observed during a six‐month follow‐up. This approach may offer a valuable therapeutic option for similar high‐risk cSCC patients.

**Key Clinical Message**

For high‐risk cutaneous squamous cell carcinoma (cSCC) complicated by myelodysplastic syndrome (MDS), combining narrow‐margin surgical excision with 5‐aminolevulinic acid photodynamic therapy (ALA‐PDT) offers an effective and well‐tolerated treatment option. This approach minimizes bleeding risk from thrombocytopenia while preventing recurrence in patients unsuitable for conventional wide excision or systemic therapies.

## 1 Introduction

Cutaneous squamous cell carcinoma (cSCC) is the second most common form of non‐melanoma skin cancer (NMSC), with a steadily increasing incidence worldwide due to factors such as ultraviolet (UV) exposure, chronic inflammation, advanced age, and genetic predisposition [1, 2]. Standard treatment for cSCC typically involves surgical excision with appropriate margins. However, when tumors are located in anatomically complex regions or in patients with compromised physiological status, alternative approaches such as photodynamic therapy (PDT) or narrow‐margin excision may be considered [3]. Myelodysplastic syndromes (MDS) are a group of clonal hematopoietic disorders characterized by ineffective hematopoiesis, peripheral blood cytopenias, and increased risk of transformation to acute myeloid leukemia [4]. Patients with MDS often present with thrombocytopenia and immunosuppression, making surgical intervention challenging due to increased risks of bleeding and infection, as well as poor wound healing.

Despite increasing knowledge regarding cSCC management being accumulated, there are few reports addressing the clinical approach to those cSCC patients with concurrent MDS. Here, we describe a cSCC patient with MDS who was successfully treated by a combination of narrow‐margin surgical excision and 5‐aminolevulinic acid PDT (ALA‐PDT), offering a potentially effective strategy for this complex clinical scenario.

## 2 Case Report

### 2.1 Presenting Complaints and Disease History

**FIGURE 1:**
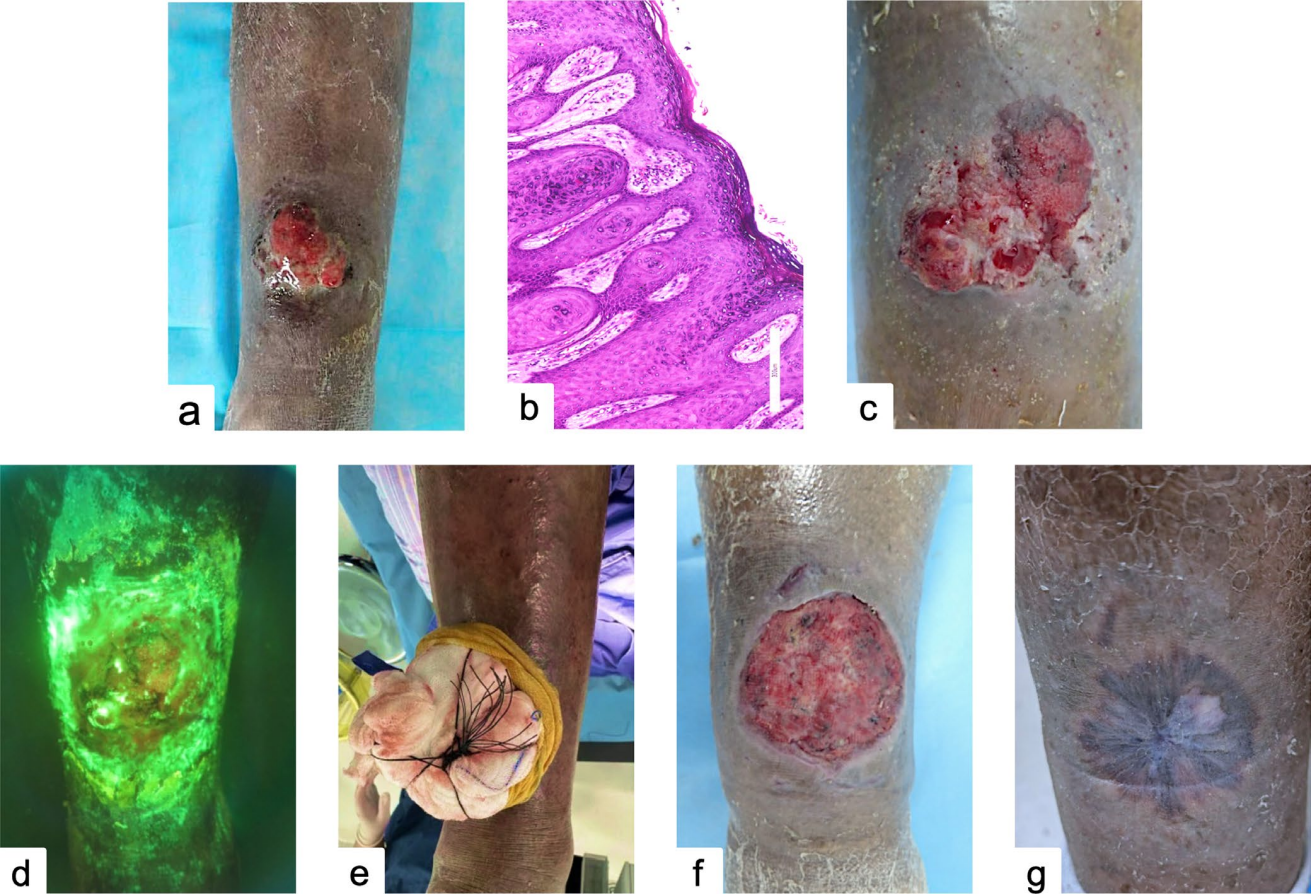
(**a**) erythematous plaque with exophytic cauliflower‐like growth. (**b**) Under high‐power magnification, squamous cell nests, keratin pearls, and inflammatory cell infiltration are observed. (**c**) Pre‐treatment view. (**d**) Wood's light reveals white fluorescence. (**e**) Postoperative photo of purse‐string suture (**f**) Post‐second ALA‐PDT. (**g**) Healed lesion after six months.

A 77‐year‐old woman presented with a gradually enlarging lesion on the anterior aspect of her right calf that had been present for over six months. Physical examination revealed a well‐defined, erythematous plaque measuring approximately 4.5 × 4.0 cm with a cauliflower‐like exophytic surface, surrounded by areas of scabbing, vesiculation, ulceration, and purulent discharge. The lesion had firm consistency, signs of basal infiltration, and was tender to palpation. No palpable lymphadenopathy was noted. Surrounding the lesion, patchy dark brown pigmentation was observed (Figure 1a). The patient's medical history was notable for a previous diagnosis of MDS made at an external hospital.

### 2.2 Differential Diagnosis and Treatment

Laboratory testing confirmed grade IV thrombocytopenia. Based on the clinical presentation and diagnostic findings, a provisional diagnosis of cSCC complicated by MDS was made. Postoperative histopathological examination revealed marked epidermal hyperplasia with invasive growth into the dermis. The tumor cells showed disordered arrangement, nuclear pleomorphism, dyskeratosis, and the formation of squamous cell clusters. Keratin pearls and squamous nests were observed, with dense peritumoral inflammatory infiltration (Figure 1b). Given the advanced age, severe thrombocytopenia, and increased surgical risk of the patient, a stepwise approach was planned using neoadjuvant PDT to reduce tumor burden and minimize intraoperative bleeding, followed by conservative surgical excision. After obtaining informed consent, an initial session of ALA‐PDT was administered (Figure 1c); Under Wood's lamp examination, the lesion showed an irregular projection with faint white fluorescence (Figure 1d). Subsequently, the tumor was completely resected under local anesthesia with purse‐string suture hemostasis (Figure 1e). Following surgical excision, additional PDT sessions were administered to the surgical site to eliminate residual malignant cells at the base and margins (Figure 1f). A 20% ALA cream (Shanghai Fudan Zhangjiang Biomedical Co. Ltd., China) was freshly prepared in the dark and applied evenly to the affected area. After 30 min of occlusive incubation in the dark, the area was illuminated with a 635 nm wavelength light‐emitting diode (LED‐IB, China) at a fluence rate of 30 mW/cm^2^ for 16 min, maintaining a distance of 10 cm. Pain was managed using a cold air analgesia system. The PDT sessions were repeated every two weeks, for a total of six times treatments. Significantly, to avoid bleeding (the primary concern) and infection, repeat biopsy was not performed in this case and PDT's therapeutic efficacy was adequately confirmed by clinical and dermoscopic evaluation.

### 2.3 Outcome and Follow‐Up

During a six‐month follow‐up, the treatment area of the patient healed well with minimal scar and without recurrence (Figure 1g). However, it was reported that the patient succumbed to an infection secondary by MDS during the 7th month of the follow‐up.

## 3 Discussion

cSCC is a prevalent form of NMSC with an increasing incidence globally, largely attributed to population aging and improved cancer surveillance efforts [5]. The standard treatment for cSCC is complete surgical excision with histologically clear margins. However, treatment may become considerably more complex when cSCC coexists with hematological disorders such as MDS [6]. MDS represents a spectrum of clonal hematopoietic disorders characterized by bone marrow dysfunction, leading to cytopenias and impaired immune responses [6]. In the context of cSCC, patients with MDS often present with significant therapeutic challenges including a high risk of bleeding due to thrombocytopenia, increased susceptibility to infection, and reduced tolerance to extensive surgical procedures. Furthermore, poor vascularization and impaired wound healing in such patients limit the feasibility of complex reconstructive surgery, particularly in anatomically constrained areas like the anterior tibial region.

ALA‐PDT has demonstrated efficacy in the management of superficial and selected invasive NMSC, including actinic keratosis, Bowen's disease, and basal cell carcinoma [7]. Its treatment mechanisms involve selective uptake of the photosensitizer in dysplastic or neoplastic cells, followed by light activation leading to localized cytotoxicity without significant damage to surrounding healthy tissue [8]. This makes it particularly suitable for elderly or immunocompromised patients who cannot tolerate more aggressive treatments. Previous reports have documented the successful use of PDT combined with surgery in treating various skin malignancies such as keratoacanthoma‐like cSCC, multiple basal cell carcinomas, and hidradenocarcinoma [9, 10, 11].

Therefore, in the present case, we adopted a multimodal treatment strategy by combining narrow‐margin surgical excision with ALA‐PDT. Firstly, preoperative ALA‐PDT served to reduce the tumor volume and vascularity, thereby minimizing intraoperative bleeding risks. Secondly, narrow‐margin excision was necessary due to the lesion's location on the anterior tibia, where excessive tissue removal or flap reconstruction was not feasible. The surgical purse‐string suture technique was effectively applied, providing circumferential wound edge approximation with uniform compression for reliable hemostasis, deep wound closure, and infection prevention, while its symmetrical tension promoted proper tissue alignment to enhance healing [12]. Postoperative ALA‐PDT was used to eliminate residual malignant cells that could remain at the surgical margins. Actually, only one study reported the treatment of a cSCC patient accompanied by MDS, which adopted the combination of the programmed death‐1 (PD‐1) inhibitor (Cemiplimab) and 5‐azacytidine, demonstrating its efficacy in controlling cSCC [13]. Besides, concerning MDS pathogenesis, they also suppose that cemiplimab in combination with 5‐azacytidine may improve MDS control. Successful outcomes were achieved through photodynamic therapy combined with narrow‐margin excision, offering a valuable alternative therapeutic approach for similar cases.

Nevertheless, the follow‐up period was relatively short (only six months) and was discontinued at seven months due to the patient's death from MDS. Therefore, longer‐term observation in similar cases remains necessary to confirm the long‐term effect of ALA‐PDT. Furthermore, the generalizability of this therapeutic approach needs validation through prospective studies with larger cohorts.

## 4 Conclusion

The combination of narrow‐margin excision and ALA‐PDT proved to be an effective and well‐tolerated strategy in treating cSCC complicated by MDS. This treatment modality may serve as an alternative for cSCC patients with contraindications to extensive surgery or systemic therapy.

## Author Contributions

**Yunwei Yang:** data curation, investigation, methodology, writing – original draft. **Zhenlin Li:** data curation, formal analysis, writing – original draft. **Long Wen:** data curation, investigation, methodology. **Yongxian Lai:** data curation, methodology. **Guolong Zhang:** funding acquisition, project administration, writing – review and editing.

## Funding

This work was supported by the National Key Research and Development Program of China, 2023YFC2508202 and the National Natural Science Foundation of China, 82272761.

## Consent

Written informed consent was obtained from the patient to publish this report in accordance with the journal's patient consent policy.

## Conflicts of Interest

The authors declare no conflicts of interest.

## Data Availability

Data sharing not applicable to this article as no datasets were generated or analysed during the current study.
